# Supplementary material for: Factors influencing catheter-related infections in peritoneal dialysis patients: a meta-analysis
Source: PeerJ. 2025 Sep 29;13:e20063. doi: 10.7717/peerj.20063 (PMC12490517; doi:10.7717/peerj.20063)
Supplement: Supplemental Information 2 [file peerj-13-20063-s002.docx]

**Appendix 1 - Database Search Process**

PubMed:

((((Peritoneal Dialysis[MeSH Terms]) OR (Continuous ambulatory peritoneal dialysis[Title/Abstract])) OR (peritoneum dialysis[Title/Abstract])) AND ((((catheter infection[MeSH Terms]) OR (catheter-related infection[Title/Abstract])) OR (catheter related infections[Title/Abstract])) OR (exit-site infection[Title/Abstract]))) AND (((risk factors[MeSH Terms]) OR (dangerous factors[Title/Abstract])) OR (influence factors[Title/Abstract]))

Cochrane:

#1 MeSH descriptor: [Peritoneal Dialysis] explode all trees 1159

#2 (Peritoneal Dialysis or Continuous ambulatory peritoneal dialysis or peritoneum dialysis):ti,ab,kw (Word variations have been searched) 2648

#3 #1 or #2 2648

#4 MeSH descriptor: [Catheter-Related Infections] explode all trees 458

#5 (Catheter infection or catheter-related infection or catheter related infections or exit-site infection):ti,ab,kw (Word variations have been searched) 5164

#6 #4 or #5 5164

#7 MeSH descriptor: [Risk Factors] explode all trees 33230

#8 (risk factors or dangerous factors or influence factors):ti,ab,kw (Word variations have been searched) 137793

#9 #7 or #8 137793

#10 #3 and #6 and #9 27

Embase:

(‘peritoneal dialysis’ OR ‘Continuous ambulatory peritoneal dialysis’ OR ‘peritoneum dialysis’):ab,ti AND (‘catheter- related infection’ OR ‘Catheter infection’ OR ‘catheter related infections’ OR ‘exit-site infection’):ab,ti AND (‘risk factors’ OR ‘dangerous factors’ OR ‘influence factors’):ab,ti

Medline:

((MHX=(Peritoneal Dialysis)) OR TS=(Continuous ambulatory peritoneal dialysis OR peritoneum dialysis)) AND ((MHX=(Catheter infection)) OR TS=(catheter-related infection OR catheter related infections OR exit-site infection)) AND ((MHX=(risk factors)) OR TS=(dangerous factors OR influence factors))

Web of Science

(Peritoneal Dialysis OR Continuous ambulatory peritoneal dialysis OR peritoneum dialysis) AND (Catheter infection OR catheter-related infection OR catheter related infections OR exit-site infection) AND (risk factors OR dangerous factors OR influence factors)

CNKI：

(Topics: Peritoneal Dialysis + Continuous Ambulatory Peritoneal Dialysis + Peritoneal Dialysis Therapy) AND (Topics: Catheter Infections + Catheter Outlet Infections + Outlet Infections) AND (Topics: Influencing Factors + Associated Factors + Risk Factors)

Wanfang：

Topic:(Peritoneal Dialysis OR Continuous Ambulatory Peritoneal Dialysis OR Peritoneal Dialysis Therapy) AND Topic:(Catheter Infections OR Catheter Outlet Infections OR Outlet Infections) AND Topic:(Influencing Factors OR Associated Factors OR Risk Factors)

VIP：

(((title or keyword=peritoneal dialysis OR title or keyword=continuous ambulatory peritoneal dialysis) OR title or keyword=peritoneal dialysis therapy) AND ((title or keyword=catheter infections OR title or keyword=infections at the exit of the catheter OR title or keyword=outlet infections) OR title or keyword=outlet infections) AND ((title or keyword=influential factors OR title or keyword=associated factors) OR Title or keyword=Risk factors)))

SinoMed:

( "Peritoneal dialysis" [common field:smart] OR "Continuous ambulatory peritoneal dialysis" [common field:smart] OR "Peritoneal dialysis therapy" [common field:smart]) AND( "Catheter infection" [common field:smart] OR "Catheter exit infection" [common field:smart] OR "Outlet infection" [common field:smart]) AND( "Influencing factors" [common field:smart] OR "Risk factors" [common field:smart]) AND( "Risk factors" [common field:smart]) AND( "Risk factors" [common field:smart] OR "Risk factors" [common field:smart]) AND( "Risk factors" [common field:smart]) AND( "Risk factors" [common field:smart] OR "Risk factors" [common field:smart]) AND( "Risk factors" [common field:smart] OR "Risk factors" [common field:smart]) Fields:Smart] OR "Associated Factors"[Common Fields:Smart] OR "Risk Factors"[Common Fields:Smart])
