# Supplementary material for: Factors influencing catheter-related infections in peritoneal dialysis patients: a meta-analysis
Source: PeerJ. 2025 Sep 29;13:e20063. doi: 10.7717/peerj.20063 (PMC12490517; doi:10.7717/peerj.20063)
Supplement: Supplemental Information 3 [file peerj-13-20063-s003.docx]

| **Table 5** **Data extracted from the literature included in the review** | | | |
| --- | --- | --- | --- |
| **variables** | **OR** | **ORLCi** | **ORUCI** |
| **Diabetes** |  |  |  |
| Xu et al., 2019 | 7.352 | 4.35 | 9.01 |
| Ding et al., 2020 | 1.631 | 1.165 | 2.284 |
| Cao et al., 2021 | 0.358 | 0.11 | 1.22 |
| Wang et al., 2021 | 3.751 | 1.593 | 7.105 |
| Huang et al., 2022 | 2.781 | 2.109 | 3.527 |
| Yang et al., 2023 | 3.467 | 1.138 | 10.567 |
| **High blood pressure** |  | | |
| Xu et al., 2019 | 1.143 | 1.050 | 1.440 |
| Wang et al., 2021 | 3.751 | 1.593 | 7.105 |
| Yang et al., 2023 | 3.705 | 1.285 | 10.680 |
| **Dialysis duration** |  | |  |
| Yue et al.,2019 | 0.099 | 0.018 | 0.539 |
| Xu et al., 2019 | 5.359 | 3.140 | 8.590 |
| Wang et al., 2021 | 5.671 | 2.376 | 5.413 |
| Wong et al.,2022 | 0.83 | 0.78 | 0.87 |
| Yang et al.,2023 | 17.642 | 5.67 | 54.892 |
| **BMI** |  |  |  |
| Xu et al., 2019 | 9.278 | 6.940 | 12.330 |
| Wang et al., 2021 | 7.297 | 3.324 | 8.715 |
| Yang et al., 2023 | 3.637 | 1.214 | 10.897 |
| **Insecure catheter placement** |  | | |
| Lin et al., 2020 | 1.770 | 1.140 | 2.750 |
| Lin et al., 2020 | 3.074 | 1.046 | 9.035 |
| Yang et al., 2021 | 1.793 | 1.06 | 3.032 |
| Yang et al., 2023 | 10.158 | 2.954 | 34.932 |
| **Mechanical strain** |  | | |
| Lin et al., 2020 | 4.920 | 2.420 | 10.000 |
| Yang et al., 2023 | 7.389 | 1.225 | 44.587 |
| **Lack of proper care** |  | | |
| Wang et al., 2021 | 6.198 | 4.289 | 9.158 |
| Yang et al., 2023 | 3.758 | 1.303 | 10.845 |
| **Irregular caregivers** |  | | |
| Lin et al., 2020 | 2.423 | 1.004 | 5.845 |
| Lu et al., 2022 | 2.989 | 1.089 | 8.206 |
| **History of catheter pulling** |  | | |
| Lin et al., 2020 | 1.780 | 1.020 | 3.110 |
| Ding et al., 2020 | 2.697 | 1.937 | 3.755 |
| Liu et al., 2021 | 5.75 | 1.878 | 17.61 |
| **Nursing process adherence** |  | | |
| Lin et al., 2020 | 2.352 | 1.008 | 5.488 |
| Ding et al., 2020 | 0.794 | 0.677 | 0.931 |
| **Serum albumin＜30g** |  | | |
| Wong et al.,2022 | 1.090 | 1.020 | 1.160 |
| Huang et al., 2022 | 7.194 | 4.872 | 9.307 |
