# Supplementary material for: Factors influencing catheter-related infections in peritoneal dialysis patients: a meta-analysis
Source: PeerJ. 2025 Sep 29;13:e20063. doi: 10.7717/peerj.20063 (PMC12490517; doi:10.7717/peerj.20063)
Supplement: Supplemental Information 4 [file peerj-13-20063-s004.docx]

**Supplementary Table S1. List of excluded studies with rationale**

| **Reference (Author, Year)** | **Study Design** | **Sample Size** | **Primary Outcome** | **Reason for Exclusion** | **Location in Screening Process** |
| --- | --- | --- | --- | --- | --- |
| Abud, A (2015) | Cross-sectional study | 416 | Peritonitis and catheter exit-site infection | Incomplete covariate data (OR) | Data extraction phase |
| Holley, J (1991) | Retrospective cohort Study | 411 | Tunnel infection | Incomplete covariate data (OR) | Data extraction phase |
| Oxton, L (1994) | Retrospective study | 163 | Catheter-related infection and peritonitis | Incomplete/Unclear Data Reporting ("New CRI" vs. "Total CRI" without explicit definitions) | Data extraction phase |
| Furth, S (2000) | Prospective cohort Study | 1258 | Catheter infections and peritonitis | No significant factors found (p>0.05 for all variables) | Full-text review |
